# Impact of high-risk of obstructive sleep apnea on chronic cough: data from the Korea National Health and Nutrition Examination Survey

**DOI:** 10.1186/s12890-022-02222-5

**Published:** 2022-11-16

**Authors:** Tae Hoon Kim, I Re Heo, Ho Cheol Kim

**Affiliations:** Department of Internal Medicine, Gyeongsang National University School of Medicine and Gyeongsang National University Changwon Hospital, 11, Samjungja-ro, Sungsan-gu, 51472 Changwon, Republic of Korea

**Keywords:** Chronic cough, Obstructive sleep apnea, Risk factors, Respiratory function tests, Obesity

## Abstract

**Background:**

Chronic cough is an extremely common clinical symptom of various diseases. However, the relationship between obstructive sleep apnea (OSA) and chronic cough in the general population has not been sufficiently studied.

**Methods:**

Using the 2019 Korean National Health and Nutrition Examination Survey data, we identified a group at high-risk of OSA via the STOP-Bang questionnaire and determined the association between OSA and chronic cough by a regression model.

**Results:**

Of the eligible 4,217 participants, 97.1% and 2.9% were classified into the non-chronic cough and chronic cough groups, respectively. The chronic cough group had higher STOP-Bang scores than those of the group without chronic cough (2.32 ± 1.38 vs. 2.80 ± 1.39; *P* < 0.001). In the group at high-risk of OSA, 40.4% and 52.0% of participants scored ≥ 3 in STOP-Bang, depending on the absence or presence of chronic cough (*P* = 0.012), respectively. Chronic cough independently correlated with impaired lung function (forced expiratory volume in one second ≥ 50–<80% predicted value, *P* = 0.001; <50, *P* < 0.001), low household income (*P* = 0.015), and a group at high-risk of OSA (STOP-Bang score 3–4, *P* = 0.004; 5–8, *P* < 0.001). Obesity I had a protective role against the occurrence of chronic cough (*P* = 0.023).

**Conclusion:**

A high-risk for OSA is a significant risk factor for chronic cough. OSA should be considered when evaluating chronic cough patients.

## Background

Chronic cough is one of the most common reasons for patients to visit respiratory clinics [1–3], and when it lasts > 8 weeks can become a serious problem [4]. It exhausts patients and adversely affects their quality of life. Doctors also face difficulties in diagnosing and treating the causes of chronic cough. Although upper airway cough syndrome, cough variant asthma, and reflux disease are the most common causes of chronic cough [5, 6], the accuracy of diagnosis should be re-evaluated based on the response to specific treatments [7]. Moreover, chronic cough could be caused by some conditions, such as various respiratory diseases, drug side effects, psychological or behavioral causes, occupational and environmental influences, peritoneal dialysis, and idiopathic causes [8]. Obstructive sleep apnea (OSA) is known to cause chronic cough [9, 10]. In particular, some studies showed an improvement in chronic cough after applying a continuous positive airway pressure (CPAP) device for the management of OSA in patients with chronic cough [11, 12].

OSA can cause several health problems, including hypertension, stroke, cardiomyopathy, heart failure, diabetes, and heart attacks [13]. Polysomnography is known as the diagnosis of choice for OSA [14, 15]; however, it requires patients to sleep overnight at the hospital and undergo assessments of electroencephalography, eyeball movements, electromyography, respiration, and electrocardiography during sleep. Limitations of time, place, or cost make it difficult to test many people. Several other attempts have been made to identify patients at high-risk of OSA [16–22], and the STOP-Bang questionnaire is simple and reliable screening tool used to identify groups at high-risk of OSA [23].

This study aimed to investigate the clinical characteristics of participants with chronic cough and determine the associated risk factors for chronic cough. Specifically, it aimed to examine the role of the high-risk of OSA in the prevalence of chronic cough in the general Korean population.

## Methods

### Data source and participants

The Korean National Health and Nutrition Examination Survey (KNHANES) is a national survey project conducted annually by the Korea Disease Control and Prevention Agency [24]. To represent the Korean population, participants were selected using a rolling sampling design that included complex, stratified, and multistage probability cluster surveys. The KNHANES data are available on the website (https://knhanes.kdca.go.kr/knhanes), and we retrospectively analyzed the survey data for 2019.

**Fig. 1:**
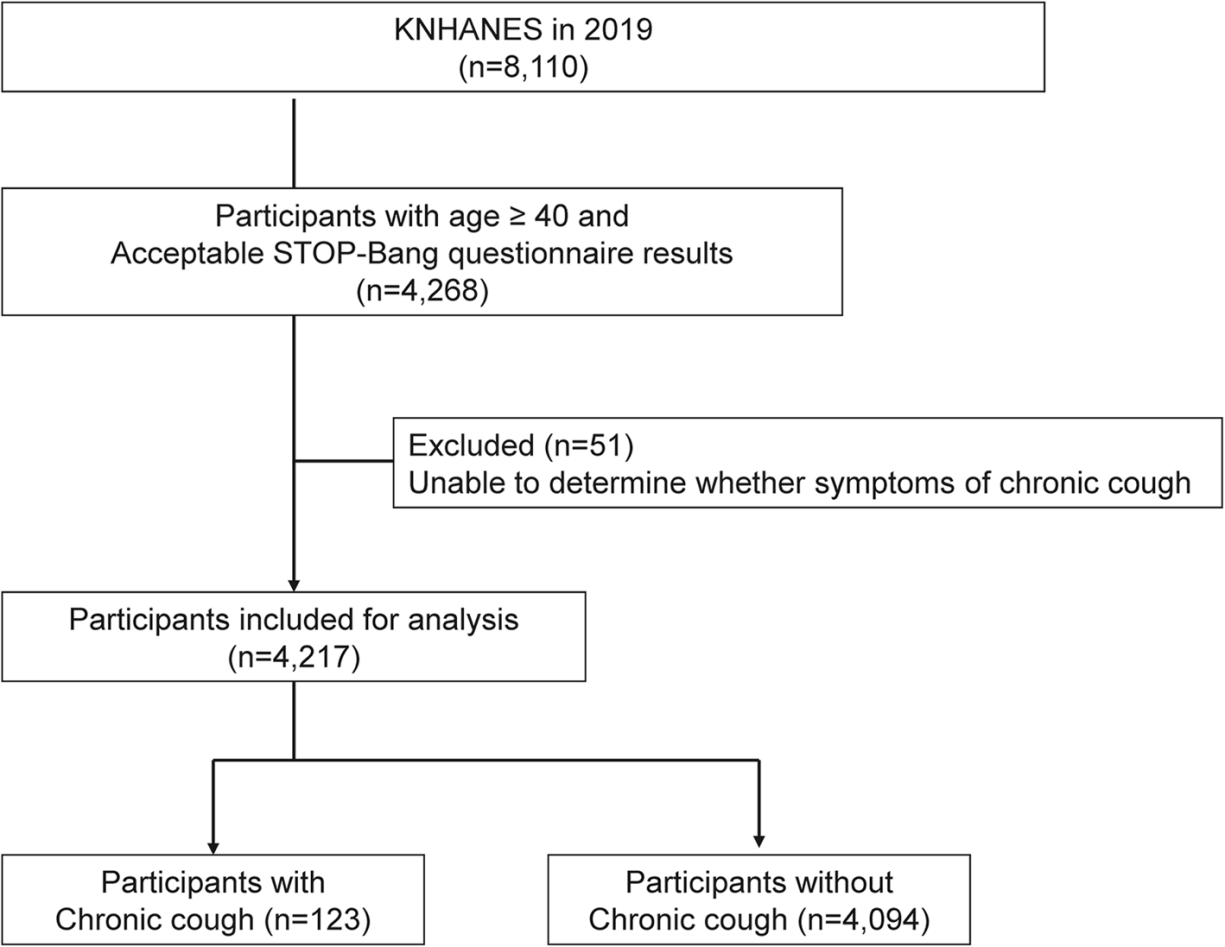
Flow diagram of participant recruitment

A flow diagram of subject recruitment is shown in Fig. 1. Among the 8,110 participants, 4,268 participants (≥40 years) with acceptable STOP-Bang questionnaire results were enrolled. However, 51 participants who could not confirm chronic cough were excluded. Finally, 123 participants who self-reported that they had a cough for over 3 months were enrolled as “participants with chronic cough.” The remaining 4,094 participants were enrolled as “participants without chronic cough.”

The KNHANES was approved by the Institutional Review Board at the Korea Disease Control and Prevention Agency, and all participants signed informed consent forms during the survey. This study was approved by the Institutional Review Board of the Gyeongsang National University Changwon Hospital (2022-04-002) and conducted according to the tenets of the Declaration of Helsinki.

### Variables

The high-risk of OSA group was assessed using the STOP-Bang questionnaire that comprises eight items: presence of snoring, tiredness or sleepiness, observed apneas or choking, hypertension, obesity (body mass index (BMI) > 30 kg/m^2^), age (> 50 years), neck circumference (> 40 cm), and sex (male). We applied the adjusted criteria for Asians in a previous study [25], and those with a total score of 3 or higher were classified as a group at high-risk for OSA. In the high-risk group, a group at extremely high-risk for OSA was defined as those with scores of 5 or higher.

Clinical demographic data, such as age, sex, BMI, smoking habit, hypertension, diabetes, and lung function, were collected. Participants were classified into four groups according to age (40–49, 50–59, 60–69, and ≥ 70 years) and five groups according to the Asian-Pacific BMI classification (underweight, < 18.5; normal weight, 18.5–22.9; overweight, 23–24.9; obese I, 25–29.9; and obese II, ≥ 25.0) [26]. Ever-smoker was defined as a person who had smoked 100 cigarettes or more in his/her lifetime. Hypertension was defined as having been diagnosed with hypertension by a physician, being treated for hypertension, or having had a measured systolic blood pressure ≥ 140 mmHg or diastolic blood pressure ≥ 90 mmHg. Diabetes was defined as a fasting blood glucose level of 126 or higher or having been diagnosed or treated by a doctor. Lung function tests were performed by trained healthcare workers using a rolling dry-seal spirometer (Vmax series 2130; SensorMedics Corp., Yorba Linda, CA, USA), according to the guidelines [27]. The past-history of asthma, allergic rhinitis, and sinusitis in the KNHANES health questionnaire was also reviewed.

Socioeconomic factors, including household income level, economic activity, and education level, were reviewed. Household income levels were classified into four categories: low income (≤ lowest quartile), middle–low income, middle–high, and high income. The economic activities of the participants were divided into employed and unemployed (or economically inactive) states. Educational level was classified as middle school or less (9 years of education or less) and high school education or higher.

### Statistical analysis

Clinical and socioeconomic demographic variables were compared between participants with and without chronic cough. Continuous variables were presented as median and interquartile ranges (IQRs), and analyzed between the two groups using the *Mann-Whitney U* test. Categorical variables, expressed as numbers and percentages of participants, were compared using the chi-squared test. Risk factors for chronic cough were analyzed using a logistic regression model. Statistically significant variables with a *P* value < 0.20 in univariate analysis were entered into the multivariate analysis. In the analysis, backward stepwise logistic regression using the likelihood ratio test was applied to determine independent factors related to chronic cough. Statistical analysis was performed using SPSS version 24.0 (SPSS Inc., Chicago, IL, USA).

## Results

### Characteristics of the study participants

The baseline characteristics of the enrolled participants are shown in Table 1. The 4,217 participants had a median age of 59.0 (IQRs, 50–69) years, of which 42.5% were male. The median BMI was 23.8 (IQRs, 21.8–26.1) kg/m^2^, and only 2.8% of all participants had a normal body weight. A total of 1,651 (39.2%) participants had an ever-smoking history, and 629 (14.9%) participants were current smokers. Of the total participants, 2.9%, 11.6%, and 5.5% had a history of asthma, allergic rhinitis, and sinusitis, respectively. Moreover, 31.8%, 26.2%, and 42.0% had normal blood pressure, pre-hypertension, and hypertension, respectively. Diabetes and pre-diabetes were observed in 18.6% and 49.1% of participants, respectively. The pulmonary function characteristics of the participants were as follows: the median forced vital capacity (FVC), forced expiratory volume in one second (FEV_1_), and FEV_1_/FVC values were 87.4 (IQRs, 79.6–95.5)% of the predicted value, 87.6 (IQRs, 79.4–95.8)% of the predicted value, and 0.78 (IQRs, 0.74–0.82), respectively. Regarding socioeconomic factors, low household income accounted for 22.9% of the total, and the unemployed or economically inactive population accounted for 41.1% of the total participants. Of the participants, 37.3% had less than 9 years of lower educational level.

**Table 1:** Baseline characteristics

Variables	Persons without chronic cough (*n* = 4,094)	Persons with chronic cough (*n* = 123)	*P* value
Age (years)			
40–49	1,025 (25.0)	21 (17.1)	<0.001***
50–59	1,098 (26.8)	15 (12.2)	
60–69	1,020 (24.9)	39 (31.7)	
≥70	951 (23.2)	48 (39.0)	
Sex			
Male	1,731 (42.3)	61 (49.6)	0.116
Female	2,363 (57.7)	62 (50.4)	
BMI (kg/m^2^)			
Underweight (<18.5)	1,508 (36.8)	48 (39.0)	0.118
Normal (18.5–22.9)	113 (2.8)	6 (4.9)	
Overweight (23–24.9)	999 (24.4)	37 (30.1)	
Obese I (25.0–29.9)	1,266 (30.9)	26 (21.1)	
Obese II (≥30)	207 (5.1)	6 (4.9)	
Smoking habit			
Never-smoker	2,493 (61.0)	64 (52.0)	
Ever-smoker (current or ex-smoker)	1,592 (39.0)	59 (48.0)	0.049*
Current smoker	592/4085 (14.5)	37/123 (30.1)	<0.001***
Hypertension			
Normal	1,312 (32.0)	30 (24.4)	0.191
Pre-hypertension	1,067 (26.1)	37 (30.1)	
Hypertension	1,715 (41.9)	56 (45.5)	
Diabetes mellitus			
Normal	1,272 (32.6)	28 (24.6)	<0.001***
Pre-diabetes	1,911 (48.9)	62 (54.4)	
Diabetes mellitus	724 (18.5)	24 (21.1)	
Asthma history			
No asthma history	3984 (97.3)	109 (88.6)	<0.001***
Asthma history	110 (2.7)	14 (11.4)	
Allergic rhinitis history			
No Allergic rhinitis history	3618 (88.4)	109 (88.6)	1.000
Allergic rhinitis history	476 (11.6)	14 (11.4)	
Sinusitis history			
No sinusitis history	3871 (94.6)	115 (93.5)	0.547
Sinusitis history	223 (5.4)	8 (6.5)	
Lung function tests			
FVC (L)	3.19 (2.65–3.83)	2.86 (2.42–3.66)	0.003**
FVC (% of predicted)	87.5 (79.6–95.6)	84.0 (75.9–92.0)	0.001**
FEV_1_ (L)	2.47 (2.06–2.95)	2.03 (1.64–2.63)	<0.001***
FEV_1_ (% of predicted)	87.7 (79.7–96.0)	80.6 (72.3–90.0)	<0.001***
≥80	2,656 (74.4)	55 (53.4)	<0.001***
≥50–<80	887 (24.8)	42 (40.8)	
≥30–<50	24 (0.7)	4 (3.9)	
<30	4 (0.1)	2(1.9)	
House income			
High	1,117 (27.4)	20 (16.4)	<0.001***
Middle–high	989 (24.2)	19 (15.6)	
Middle–low	1,058 (25.9)	36 (29.5)	
Low	916 (22.5)	47 (38.5)	
Economic activity			
Employed	2,431 (59.4)	53 (43.1)	<0.001***
Unemployed, inactive	1,662 (40.6)	70 (56.9)	
Educational level			
High (>9years)	2,576 (63.0)	66 (53.7)	0.038*
Low (≤9 years)	1,515 (37.0)	57 (46.3)	

Data are presented as number (%) or median (interquartile ranges), unless otherwise indicated

*Abbreviations*: *BMI* body mass index, *FVC* forced vital capacity, *FEV*_*1*_ forced expiratory volume in 1 s

*P < 0.05, **P < 0.01, ***P < 0.001

Patients in the chronic cough group were older than those in the group without chronic cough (*P* < 0.001). There was no difference in the distribution of sex, BMI, and prevalence of hypertension between the two groups (*P* > 0.05). Participants with a chronic cough had a higher smoking history (*P* = 0.049) and a higher prevalence of diabetes (*P* < 0.001). A history of asthma was more common in the chronic cough group (*P* < 0.001), but the frequencies of allergic rhinitis and sinusitis did not differ between the two groups. Overall, compared with 74.4% of participants without chronic cough, only 53.4% of participants with chronic cough had normal lung function (*P* < 0.001). Participants with chronic cough had a lower educational background (*P* = 0.038), household income level (*P* < 0.001), and economic activity (*P* < 0.001) than those without chronic cough.

### Group at high-risk of OSA

**Fig. 2:**
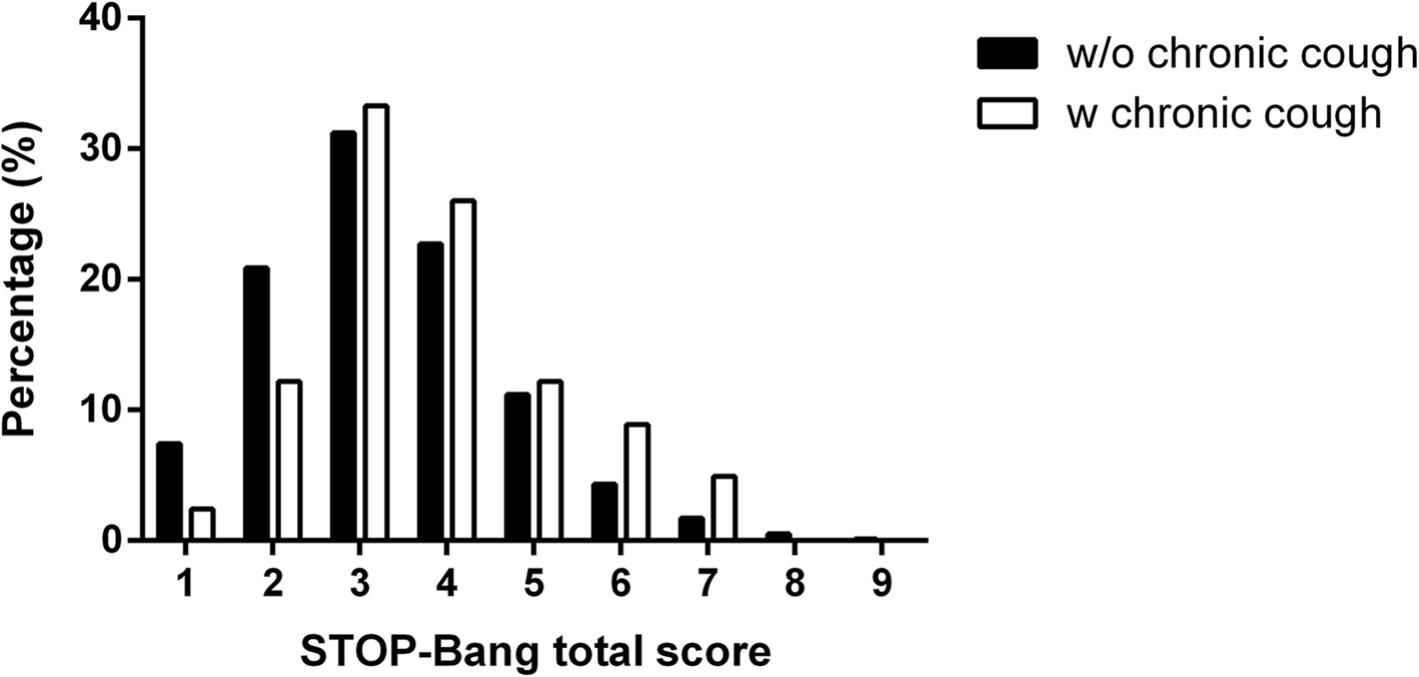
Distribution of total STOP-Bang questionnaire score

The distribution of the STOP-Bang questionnaire results is shown in Fig. 2; Table 2. The total score of the STOP-Bang questionnaire was median 2.0 (IQRs, 1.0–3.0; mean + standard errors, 2.33 ± 1.38) and the chronic cough group had a higher score than the other group without chronic cough (*P* < 0.001). The group at high-risk of OSA, with a score of 3 or higher, comprised 52% and 40.4% of all participants, depending on the presence or absence of chronic cough (*P* = 0.012). In participants with chronic cough, the frequency of group at extremely high-risk for OSA was higher than in participants without the symptom (*P* = 0.005).

**Table 2:** Group at high-risk for OSA by the STOP-BANG questionnaire

STOP-BANG questionnaire variables	Persons without chronic cough (*n* = 4,094)	Persons with chronic cough (*n* = 123)	*P* value
STOP-Bang			
Total score (0–8)	2.0 (1.0 – 3.0)	3.0 (2.0 – 4.0)	<0.001***
	2.32 ± 1.38^a^	2.80 ± 1.39^a^	<0.001***
Group at high-risk of obstructive sleep apnea			
≥3 points in STOP-Bang	1,656 (40.4)	64 (52.0)	0.012*
≥5 points in STOP-Bang	270 (6.6)	17 (13.8)	0.005**

Data are presented as median (interquartile ranges), unless otherwise indicated

*P < 0.05, **P < 0.01, ***P < 0.001

^a^number (%) or mean ± standard deviation

Among the eight items of the STOP-Bang questionnaire, the presence of snoring, tiredness (or sleepiness), and older age (over 50 years old) showed differences between the groups with and without chronic cough. Persons with these three items were more common in the chronic cough group. However, there were no significant differences in the observed apneas (choking), hypertension, obesity, thick neck circumference (> 40 cm), and sex.

### Association between high-risk of OSA and chronic cough

The risk factors associated with chronic cough are shown in Table 3. In the univariate analysis, old age (60–69 years, *P* = 0.023; ≥70, *P* = 0.001), current smoking (*P* < 0.001), asthma history (*P* < 0.001), poor lung function (FEV_1_ ≥ 50–<80, *P* < 0.001; <50, *P* < 0.001), low household income (*P* < 0.001), low-grade educational level (*P* = 0.036), reduced economic activity (*P* < 0.001), and an extremely high-risk of OSA (*P* = 0.001) were associated with chronic cough. There was no relationship between chronic cough and sex, BMI, history of allergic rhinitis or sinusitis, hypertension, or diabetes.

**Table 3:** Risk factors for chronic cough

Variables	Categories of variables	Univariate analysis		Multivariate analysis	
		Exp. (95% CI)	*P* value	Exp. (95% CI)	*P* value
Age (years)	40–49	1.000		1.000	
	50–59	0.667 (0.342–1.301)	0.234	0.594 (0.284–1.244)	0.167
	60–69	1.866 (1.090–3.195)	0.023*	1.315 (0.673–2.567)	0.423
	≥70	2.464 (1.464–4.145)	0.001**	2.396 (1.192–4.816)	0.014*
Sex	Female	1.000		1.000	
	Male	1.343 (0.938–1.923)	0.107	0.603 (0.349–1.042)	0.070
BMI (kg/m^2^)	Underweight (<18.5)	1.668 (0.699–3.982)	0.249	1.727 (0.613–4.865)	0.301
	Normal (18.5–22.9)	1.000		1.000	
	Overweight (23–24.9)	1.164 (0.752–1.800)	0.496	0.925 (0.548–1.559)	0.769
	Obese I (25.0–29.9)	0.645 (0.398–1.046)	0.075	0.517 (0.293–0.913)	0.023*
	Obese II (≥30)	0.911 (0.385–2.154)	0.831	0.549 (0.194–1.557)	0.260
Smoking habit	No-smoker	1.000		1.000	
	Current-smoker	2.539 (1.710–3.768)	<0.001***	3.080 (1.829–5.186)	<0.001***
Hypertension	Normal	1.000		1.000	
	Pre-hypertension	1.517 (0.931–2.471)	0.095	1.190 (0.674–2.099)	0.549
	Hypertension	1.428 (0.911–2.238)	0.120	0.591 (0.318–1.096)	0.095
Diabetes mellitus	Normal	1.000			
	Pre-diabetes	1.474 (0.938–2.316)	0.092		
	Diabetes mellitus	1.506 (0.866–2.617)	0.147		
Asthma history	No asthma history	1.000		1.000	
	Asthma history	4.652 (2.584–8.374)	<0.001***	2.606 (1.124–6.042)	0.026*
Allergic rhinitis history	No AR history	1.000			
	AR history	0.976 (0.555–1.717)	0.934		
Sinusitis history	No sinusitis history	1.000			
	Sinusitis history	1.208 (0.582–2.504)	0.612		
FEV_1_ (% of predicted)	≥80	1.000		1.000	
	≥50–<80	2.287 (1.519–3.441)	<0.001***	1.936 (1.249–3.001)	0.003**
	<50	10.348 (4.119–25.999)	<0.001***	5.327 (1.853–15.314)	0.002**
House income	High	1.000			
	Middle–high	1.073 (0.569–2.022)	0.828		
	Middle–low	1.900 (1.093–3.304)	0.023*		
	Low	2.866 (1.686–4.871)	<0.001***		
Educational level	High (>9years)	1.000			
	Low (≤9 years)	1.468 (1.024–2.105)	0.036*		
Economic activity	Employed	1.000		1.000	
	Unemployed, inactive	1.932 (1.345–2.775)	<0.001***	1.660 (1.047–2.633)	0.031*
STOP-Bang	0–2	1.000		1.000	
	3–4	1.401 (0.950–2.067)	0.089	2.230 (1.251–3.975)	0.007**
	5–8	2.602 (1.495–4.527)	0.001**	6.614 (2.816–15.537)	<0.001***

*Abbreviations*: *AR* allergic rhinitis, *BMI* body mass index, *FEV*_*1*_ forced expiratory volume in 1 s

*P < 0.05, **P < 0.01, ***P < 0.001

Subsequently, multivariate analysis showed that advanced age (≥ 70 years, *P* = 0.014), current smoking status (*P* < 0.001), asthma history (*P* = 0.026), poor lung function (FEV_1_ ≥ 50 to < 80, *P* = 0.003; <50, *P* < 0.002), impaired economic activity (*P* = 0.031), and a high-risk of OSA (STOP-Bang score 3–4, *P* = 0.007; 5–8, *P* < 0.001) were associated with chronic cough (Nagelkerke-*R*^2^: 0.124, Hosmer–Lemeshow Goodness-of-fit: *P* = 0.281). Conversely, obesity I showed a negative relationship with the occurrence of chronic cough (*P* = 0.023). The risk-adjusted analysis showed that household income and education levels lost their relationship with chronic cough.

## Discussion

We determined the prevalence of chronic cough to be 2.9%, which is lower than that reported in previous studies. Globally, the prevalence of chronic cough varies from 2 to 18% [28]. The reason for such a large deviation was the variability of the definition of chronic cough as well as the different distribution of age in each study [29]. In the present study, cough lasting for more than 3 months was defined as a chronic cough. This is the most commonly applied definition in previous studies [28]. However, in other studies, chronic cough was also defined as cough for more than 2 months, cough for more than 3 months for two consecutive years, and cough and phlegm for more than 3 months for two consecutive years. Another study reported an increase in prevalence according to Caucasians or region of origin [29], but we investigated only the general Korean population. A previous study using 2010–2012 KNHANES data reported that the prevalence of chronic cough in Korea as 2.6% [30], similar to our results. Therefore, the prevalence of chronic cough in the general Korean population was lower than that in other countries.

The prevalence of OSA, a cause of chronic cough, is difficult to determine in patients with a chronic cough. According to a recent European study, 5.4% of the chronic cough group self-reported that they had OSA, which was significantly higher than 1.9% of participants who did not complain of chronic cough symptoms [31]. We showed that 52.0% of the patients in the chronic cough group were classified as a group at high-risk for OSA. The proportion of patients with a high-risk of OSA was significantly higher than that of patients without a chronic cough.

Meanwhile, 40.8% of the enrolled participants were identified as high-risk for OSA. Jeon et al. showed that the group at high-risk of OSA was 30.8% of the total participants in a study conducted at a hospital in Korea [25]. Compared to our study, the study enrolled young adults (≥20 years), and the sample size was small. They also reported that the STOP-Bang questionnaire had a higher diagnostic performance in OSA screening than the Berlin Questionnaire or the four-variable screening tool. Based on a preliminary study, the items of the STOP-Bang questionnaire were included in the 2019 KNHANES for the first time by the Korea Disease Control and Prevention Agency. Therefore, this study was the first to determine the frequency of individuals at high-risk for OSA in the general Korean population.

Previous studies have shown that the prevalence of chronic cough is significantly increased in the elderly [29, 31–33]. In a study on the general Korean population, age over 65 years was also reported as one of the risk factors for chronic cough [30]. In the present study, univariate analysis also showed an increased risk of chronic cough in the elderly group aged 60 years or older, and statistical significance was maintained in the risk-adjusted analysis for those aged ≥70 years.

We showed that economic activity is an independent risk factor for chronic cough. It was found that unemployed or non-job seekers were more vulnerable to chronic coughs than those working. However, household income and educational level were not risk factors for chronic cough in this study. There are various reports on the role of socioeconomic factors in chronic cough. A recent study found that low socioeconomic status contributed to an increased prevalence of chronic cough [31, 34]. In a study of young adults in Italy, socioeconomic status according to occupation had an effect on the prevalence of chronic cough [35]. In a German study, neither income nor education level affected the prevalence of chronic cough; however, as in our study, employment status was associated with chronic cough [32]. However, blue-collar occupation or high household income had no significant effect on chronic cough in another study [30].

We demonstrated that airflow limitation is one of the most powerful risk factors for chronic cough. In particular, participants with less than 50% of the predicted value of FEV_1_ showed a five-fold higher risk of chronic cough. According to a recent Canadian study, the incidence of chronic cough was higher at a lower predicted value of FEV_1_ [29]. Furthermore, participants with a history of asthma had more complaints of chronic coughing. This pattern was consistent with the findings of previous studies. In studies in Austria, Germany, Canada, the Netherlands, Finland, Denmark, and Korea, asthma has been identified as an important association with an increase in chronic cough [29–33, 36, 37]. Recent systematic reviews have shown that asthma is a strong risk factor for chronic cough [34].

Epidemiological studies have shown that obesity is associated with chronic coughs [38–40]. Indeed, chronic cough in patients with asthma and reflux disease plays an essential role in the onset and severity of symptoms and treatment response. However, in the present study, participants belonging to obese class I had a significantly lower risk of chronic cough. It is difficult to explain the exact mechanism or cause, but there is a phenomenon in which obesity causes profit, as in the present study. In some cardiovascular diseases, overweight or obese patients may have a better prognosis; this phenomenon is known as the “obesity paradox” [41]. They suggested that cardiorespiratory fitness is a more fundamental factor than BMI is. Further, the evaluation of body composition compartments and the presence of metabolic derangements may be better indicators of cardiovascular disease risk than BMI alone.

In the present study, we showed that chronic cough was three times more common in current smokers. Several previous studies have also reported that current smoking is associated with chronic cough [29, 32, 34–36]. Chronic bronchitis is the most common cause of coughing in smokers is known as chronic bronchitis [42]. Smoking cessation is the most effective treatment for coughing due to chronic bronchitis, which improves cough in approximately 90% of patients [43, 44].

### Limitations

Our study had some limitations. As it was conducted in the general population across the country, OSA could not be diagnosed using polysomnography. Since the polysomnography test is expensive and time-consuming, the group at high-risk for OSA was classified using the STOP-Bang questionnaire and its association with chronic cough was analyzed. Therefore, there may be limitations in determining the relationship between OSA and chronic cough. It is also difficult to determine whether a cause-effect relationship exists. Due to the COVID-19 pandemic, lung function tests that could cause droplet infection and concurrent questions about chronic coughs were excluded from the 2020 KNHANES. Therefore, the analysis was reduced to a one-year cross-sectional study. There were no data on past history or current prevalence of reflux disease in the 2019 KNHANES. Therefore, an analysis of reflux disease and chronic cough, a common cause of chronic cough, was not conducted. In addition, the 2019 KNHANES do not have sufficient data on respiratory illness (ex. bronchiectasis, interstitial lung disease), the full history of taking medicine, and past occupational exposure history, because it is a study with the general population. The chronic cough group accounted for only 2.9% of the total participants, and the size of the two groups was asymmetry. This imbalance may have influenced the study results. Nevertheless, this study has high reliability and representativeness as it uses data from a national survey conducted by a national institution.

## Conclusion

Chronic cough accounted for 2.9% of the total number of participants. In the participants with chronic cough, the group at high-risk for OSA was confirmed to be 52%, which is significantly higher than 40.4% of those who did not complain of chronic cough. In the risk-adjusted analysis, advanced age, current smoker, asthma history, impaired lung function, inactive economic activity, a high-risk for OSA, and obesity class I (negative correlation only) were identified as factors related to chronic cough. Therefore, when assessing patients with chronic cough, it is necessary to check whether they are at high-risk for OSA or exhibit symptoms of OSA.

## Data Availability

The datasets used and/or analyzed during the current study are available from the corresponding author on reasonable request. And, raw database of the KNHANES is available on the Korea Disease Control and Prevention Agency website (https://knhanes.kdca.go.kr/knhanes).
